# Influence in everyday life is limited by institutional cultures: A photo elicitation interview study with frail older persons in Swedish residential aged care facilities

**DOI:** 10.1371/journal.pone.0319059

**Published:** 2025-02-28

**Authors:** Roar Hermansen Østby, Synneve Dahlin-Ivanoff, Sara Hultqvist, David Edvardsson, Qarin Lood

**Affiliations:** 1 Department of Health and Rehabilitation, Institute of Neuroscience and Physiology, Sahlgrenska Academy, Centre for Ageing and Health—AgeCap, University of Gothenburg, Gothenburg, Sweden; 2 Centre for Person‑Centred Care (GPCC), University of Gothenburg, Gothenburg, Sweden; 3 Department of Psychiatry and Neurochemistry, Institute of Neuroscience and Physiology, Sahlgrenska Academy, Centre for Ageing and Health—AgeCap, University of Gothenburg, Gothenburg, Sweden; 4 Faculty of Social Sciences, School of Social Work, Lund University, Lund, Sweden; 5 School of Nursing and Midwifery, Faculty of Health Sciences, La Trobe University, Melbourne, Victoria, Australia; 6 Sahlgrenska Academy, Institute of Health and Care Sciences, University of Gothenburg, Gothenburg, Sweden; Vrije Universiteit Brussel, BELGIUM

## Abstract

Earlier studies highlighted the significance of engaging in preferred activities to maintain health and well-being after moving into a residential aged care facility. However, there seem to be significant limitations in the range of activities available, often failing to meet frail older persons’ preferences and needs This raises concerns about the adequacy of opportunities for persons living in residential age care facilities to engage in everyday activities and maintain a sense of purpose and well-being. This study aimed to identify and elucidate frail older persons’ opportunities to influence their everyday life in residential aged care facilities. Thirteen frail older persons living in residential aged care facilities were involved by being asked to take photos of their everyday life over the course of one week. Photo-elicitation interviews were then conducted together with a researcher, to narrate the content and meaning of the photos in relation to opportunities to influence their everyday life. The interviews were analysed using reflexive thematic analysis. The analysis generated one overarching theme: *Influence in everyday life is limited by institutional cultures,* illustrating how the facilities functioned as institutions that ruled the older persons’ everyday lives. This is further described in two interrelated core themes*: 1) A game of power between older persons and staff* with the sub-themes *Feelings of inferiority* and *Infringement of integrity*, and 2) *The importance of dialogue,* with the sub-themes *The surrounding as a catalyst for the person’s life-story* and *Becoming part of a community.* Institutional cultures were described to affect everything that occurred in the residential aged care facilities, which meant that the older persons’ preferences and needs were not always met. There is a need to further explore the opportunities for persons living in residential aged care facilities to participate in studies on how to attend to their wishes and needs.

## Background

As many societies over the world face the outcomes of an ageing population, the World Health Organization (WHO) [1] has listed five key strategies for ageing and health, in line with the United Nations [2] Sustainable Development Goals (SDG). This is an important step for furthering inclusion, equality, and equity. For persons living in residential aged care facilities in Sweden, this has implications on the opportunities to live a dignified life with self-determination and safety, as described in the Swedish Social Services Act [3], the Healthcare Act [4], and the Patient Act [5]. It is in the light of current Swedish legislation and SDG three and 10 (Good health and wellbeing, and Reduced inequalities) [2] this study set out to involve persons living in residential aged care facilities in the research process to identify issues of importance for further research on ageing and health. The study is part of a larger project with focus on involvement of frail older persons in research. It is connected to a previous publication by the authors [6], that aimed to evaluate photo-elicitation interviews as a participatory method to explore everyday life in residential aged care facilities. Photo-elicitation interviews proved to be a promising method to involve persons living in residential aged care facilities in the research process [6], which is why we in this publication put focus on the content and meaning of the photos taken by the older persons during the study.

Swedish aged care is primarily grounded on the policy of ageing in place [7], meaning that older persons’ care needs are adhered to in ordinary housing for as long as possible. When remaining in ordinary housing is no longer possible, residential aged care facilities are provided by the Swedish society after an assessment of each person’s needs. To be granted an apartment in a residential aged care facility, the person needs to be 65 years of age or older and have an impaired ability to orient themselves in the environment or being in intermittent need of care and supervision [8,9]. To live in a residential aged care facility is thus often associated with frailty, illness, dependency in everyday activities, and/or insufficient support to fulfill care requirements within the community. As described by Fried et al. [10], frailty can be defined as a health state continuum related to ageing. This means that as physiological capacity diminishes with age, the ability to resist functional decline and morbidity also decreases [11]. In addition, previous studies suggest that more than two thirds of the persons living in Swedish residential aged care facilities suffer from neurocognitive disorders [12]. Consequently, older persons living in residential aged care facilities often require tailored support in everyday activities, especially as their functional abilities decline [13,14].

Everyday life comprises activities people do to get a sense of meaning in life [15] and feel connected to who they are [16,17]. Earlier studies in residential aged care facilities have highlighted the significance of engaging in preferred activities to uphold one’s sense of identity and belonging [18,19]. However, influence on everyday activities is not an axiom in residential aged care facilities that often have a top-down perspective with regards to how everyday life is planned and organised [20,21]. For frail older persons, issues may also arise when there is a discrepancy between how everyday activities are currently performed, compared to how they were done when the person was younger and healthier [22]. Keane et al. [23] suggest further investigations of older persons’ preferences and needs to engage in meaningful activities and how this relates to the actual opportunities they have to choose which activities to engage in when living in a residential aged care facility [23]. Addressing the gap between older persons’ needs for meaningful activities and the limited options typically available in residential aged care facilities [24,25], this study was therefore designed to provide insights into how to promote health and well-being through involvement of older persons living in residential aged care facilities in everyday life.

Person-centred care (PCC) has been described as an ethical approach to care [26] that can promote the opportunities for older persons with neurocognitive disorders to be involved in everyday activities in residential aged care facilities [27]. It has also been described as an approach that aims to foster continuity of self and a sense of normalcy for the older person in need of care [28]. As such, it is often used as a measure of quality residential aged care [29]. Nevertheless, previous research in residential aged care facilities indicates that PCC is not always a natural part of everyday life in residential aged care facilities. There are reports on difficulties for persons living in residential aged care facilities to engage in activities that they prefer and/or need to do [16,20], as well as a documented limitation with regards to the range of activities offered. Activities may be basic or stereotypical, such as board games or crafts [30], which means that persons living in residential aged care facilities may be deprived of opportunities to engage in activities that could promote their health and well-being [31,32]. Such deprivation could have a negative impact on a persons’ well-being and quality of life [29,33] relating to feelings of boredom, frustration, isolation and even a loss of identity. Over time, this can lead to decline in the person’s both physical and mental health, as well as reduced sense of purpose and satisfaction in life [14,34]. With knowledge on what might facilitate or hinder older persons’ engagement in everyday activities, health and social care professionals can work to promote the dignity and overall well-being of frail older persons living in residential aged care facilities despite any limitations they may face [35,36]. Yet, little is known here on how to succeed with this mission and support engagement in meaningful everyday activities in residential aged care facilities [20,21,23,37]. With the goal to expand the opportunities of older persons living in residential aged care facilities to engage in everyday activities, this study therefore aimed to identify and elucidate frail older persons’ opportunities to influence their everyday life in residential aged care facilities.

## Methods

Recognising that persons outside academia are experts on their own experiences and understand best what is needed to enhance their lives, this study used photo-elicitation interviews [38] as a participatory research method with 13 persons living in two different residential aged care facilities in a middle-sized Swedish city. One of the facilities housed up to 64 persons and was specialised in care with people living with neurocognitive disorders (mild to major), according to Diagnostic and Statistical Manual of mental Disorders 5 (DSM5) [39]. The other facility housed up to 102 persons and had no specific specialisation.

The use of photographs as a method for capturing moments, surroundings and/or situations, which are important to the person taking the picture, has been applied in research for a long time. The approaches have, however, been different and have different names like photovoice, participatory photographic research, participant photography and photo-elicitation [40]. The present study used participant driven photo-elicitation interviews as described by Harper [38], as persons who live in residential aged care facilities may need visual reminders to narrate their opportunities to influence everyday life. Thus, even if the narratives in the method may consist of both photos and dialogues [38], the photos taken as part of this study functioned as a catalyst for dialogues on the meaning of the motifs [6] and were therefore not separately analysed. Since the photos were taken in the older persons’ homes, they are not included in this publication due to ethical considerations to certify the older persons’ anonymity and confidentiality. To provide an overview of what the older persons photographed, we have compiled information on the photos in Table 1. Note that all names are fictitious and randomly assigned.

**Table 1:** Descriptive information on photos taken by the involved persons.

	Age (years)	Gender	Took photos on/of:
**Alice**	91	Woman	People sitting at a nicely set dining table at a celebration The facility convenience store (cupboard with glass doors)
**David**	84	Man	A wall-mounted clock and glasses
**Emma**	85	Woman	Towels laying on the floor A dressing-gown laying on the bed
**Lisa**	89	Woman	An alarm button on the wrist A pen laying on the floor Open doors A table with bingo accessories.
**Maria**	94	Woman	Bingo accessories A coffee cup An alarm button A single crutch Papillotes
**Sara**	92	Woman	A table and chairs in an empty dining room
**Julia**	94	Woman	Today’s menu Newspapers An open book
**Victoria**	78	Woman	A sliding door to the toilet Extension cords laying around on the floor Several charging iPads
**John**	83	Man	The outdoor environment, with nature A colourful painting Differently shaped rocks in a box
**Olivia**	87	Woman	Two persons sitting in an armchair A person reading a paper in an armchair Two persons standing by a bed
**Sofia**	84	Woman	A person sitting on a bed A person holding another person’s shoulder A house Several people in a room with face masks A person with a guitar
**Thomas**	79	Man	Postcards A notebook A closed door Armchairs with and without people sitting in them A coffee cup A decorative plate on the wall The facility garden with a tree and gazebo
**Hanna**	71	Woman	People sitting in armchairs The parking lot Colourful flowers A TV Two muffins on a plate A self-portrait in a mirror

### Ethics approval and consent to be involved

This study received ethical approval from the Swedish Ethical Review Authority (Dnr. 813-18 and 2019-03112) and has been carried out in accordance with the Declaration of Helsinki. All participants received written and verbal information about the study and were informed about the possibility to withdraw their consent to be involved anytime without questions or impact on their care in the residential aged care facility. In addition, as participants used a camera in this study, they were specifically informed about the ethics of photographing other people (including residential aged care professionals).

### Involvement

To get in contact with persons living in the residential aged care facilities, professionals working in the facilities distributed information about the study’s aim and conduct, i.e., what would be required of them should they choose to be involved in the study. The information was given both verbally and in writing to eligible persons by either residential aged care professionals or the last author. Eligibility criteria were as follows: assessed by residential aged care professionals as A) having sufficient cognitive ability to give informed consent, B) being able to hold a conversation for at least 15 minutes. The time period of 15 minutes was chosen to be able to generate rich, detailed and meaningful data. Since the study was conducted during the COVID-19 pandemic, restrictions regarding external visitors were in place alongside cancelled organised activities and physical distancing. In Sweden, no visitors were allowed in residential aged care facilities, and the first eight persons were therefore involved by persons who worked there and knew the older persons well. They were, however, not involved in the older persons’ direct care. When the restrictions were lifted, the last author was able to visit the larger facility to interview the first eight persons, and the smaller facility to invite an additional seven persons to be involved in the study. Two of the additional seven persons refrained from being involved in the study due to not feeling comfortable taking responsibility for a camera not belonging to them. This means that a total of 13 persons were involved in the study. They were between 71 and 94 years of age, ten of them were women and three were men.

### Data generation

Data were generated between November 2020 and November 2021. After written consent to be involved had been received, the involved persons were instructed by residential aged care professionals (the first eight persons) or the last author (the additional five persons involved) to use a digital polaroid camera (provided to them by the researchers) to document aspects of relevance for their everyday life in the residential aged care facility. This could be objects, situations, or places they thought were concerning or important. They were in possession of the camera between two and seven days and there were no instructions on how many photos they should take. Most of the older persons requested assistance from residential aged care professionals to assist them with taking the photos, however without involvement in what to take photos of. Following the photographing period, an interview with the last author was scheduled to be conducted in each person’s apartment. Because of COVID-19 restrictions on external visitors, the first eight interviews were conducted by the last author approximately six weeks after the photos were taken (when the restrictions were lifted). The remaining five interviews were conducted by the last author promptly after the older persons had taken their photos.

The interviews were conducted in the form of dialogues and lasted between 17 and 70 minutes. During the interviews the older persons were encouraged to express their thoughts when they took the photos and why they chose the photographed motif. All interviews started with an introduction to the study, followed by the question: “Which photos do you want to talk about?” Followed by the question: “Can you describe what is on the photo?”. After the initial dialogue on what was on the photos the older persons had chosen, they were asked “What does the motif mean to you?” and “What were your thoughts when taking the photo?”. In total, 80 photos were taken, distributed from one to ten photos per person. The participant selected a number of photos they wanted to talk about, and these photos were printed during the interviews. Most of the older persons chose to keep the photos (copies were then printed for research purposes). The interviews were recorded digitally and transcribed verbatim. In addition, field notes were taken during involvement, in conversations with the persons who had been responsible for involving the older persons. The purpose of the field notes was to deepen the understanding of the context in which the photos had been taken. The consent forms, photos, recordings and transcripts are stored at the University of Gothenburg in a secure and locked cabinet, which only the involved researchers have access to. Photos, audio-recordings, and transcripts were de-identified and shared among the researchers involved in the study.

### Data analysis

The analysis was performed with reflexive thematic analysis (RTA) described by Braun and Clarke [41–43]. Regarding reflexivity we take a stance in that “…research can be described as the use of a critical, self-aware lens to interrogate both the research process and our interpretation or representation of participants’ lives in our social world” [44, p.120]. That is, to reflect on the data knowingly and consciously, to ensure that the analysis is created in the “… intersection of data, analytic process and subjectivity” [45, p.594]. The study was conducted inductively, through the different lenses of the authors who have different professional backgrounds (occupational therapy, nursing, social work) and are involved in different research settings. The study is situated within the constructivistic paradigm and is inspired by an interpretivist epistemology, reflecting the aim and research questions we sought to address. The diverse backgrounds of the authors (registered occupational therapists (RHØ, SDI, QL), social worker (SH) and registered nurse (DE)) with experiences from research on ageing and health as well as person-centred care brought different clinical and theoretical perspectives to the analysis. As such, the reflexivity in the analysis meant that the researchers reflected on what the data means and their role in the interpretation in every step of the analysis to allow for reflection on their positions and how the subjectivity may influence their processing of the data [43].

The first author was responsible for the analysis of the data. NVivo 12™ were used for data organisation, and further processed by the first author. No automatic NVivo 12™-induced analysis was executed. Quotations are presented to animate the text, as suggested by Eldh et al. [46].

### Analysis process

Braun and Clarke [41–43] describe six phases of RTA. In the first phase we familiarised ourselves with the data. All authors listened to at least two of the interviews, and the first, second and last author listened to all interviews and read all transcripts. The first author read the transcripts several times and took a first glance of the photos. In phase two, one transcript at a time was re-read by the first author, with the study aim as a beacon, and notes were taken to generate initial codes. The codes were discussed with the other authors, and during phase three, initial themes (candidate themes) were generated from the collated codes. Phase four consisted of further reviews, discarding, merging, and evolving the themes in discussions amongst all authors. In phase five, the first author further developed and determined the narrative of each theme. During phase six of the analysis was finalised by joint writing of the results by all authors [41–43].

## Results

### Influence in everyday life is limited by institutional cultures

**Fig 1:**
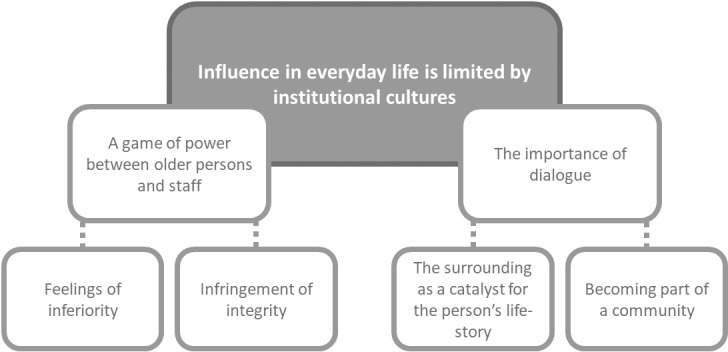
Overview of the thematic structure.

The analysis resulted in the overarching theme: *Influence in everyday life is limited by institutional cultures,* which meant that the residential aged care facilities functioned as institutions with rules, norms, and routines that influenced both what the older persons did and how they experienced their everyday lives. Everyday life in the facilities was affected by a disharmony between the older persons’ needs and the routines of the facility. How this relates to opportunities to influence everyday life is further described in two core themes: *A game of power between older persons and staff,* and *The importance of dialogue,* both with two subthemes each (Fig 1). The core themes are a dynamic whole, and to be regarded as two sides of the same coin. When dialogue was more prominent, the power was described to shift towards the older person and when power was more prominent, opportunities for dialogue diminished.

#### A game of power between the older persons and staff.

The game of power between the older persons and staff was described as staff exercising their power to govern everyday routines in the facility, and the older persons exercising their power of sharing or withholding information to feel in control. Even if the older persons had to adapt to staff routines, they strove to make their voices heard (visualised by the quotation below) and express their needs to maintain self-determination and power over their situation. For instance, some older persons initiated their own routines, such as walking to the post box several times per day to meet people and get a walk, to contrast staff routines and create meaning in everyday life. The game of power between older persons and staff is further described in the subthemes: *Feelings of inferiority*, and *Infringement of integrity*.

*“Alice: Suddenly, I didn’t get any, so I went out and said, why haven’t I received any medicine? ‘Yes, you have’. No, I haven’t received any. Then they looked in the binder, and I hadn’t received any.”*

Alice, 91 years

***Feelings of inferiority:*** The game of power and the power position of staff manifested itself through the older persons’ feelings of inferiority within the institutional cultures, meaning that they, at times, neither felt like an equal partner, nor as part of a community. They described that they sometimes felt as if they were treated as objects, or tasks, rather than as equal and respected persons. Feelings of inferiority further affected the older persons’ opportunities to influence their everyday life negatively in terms of feeling excluded and unimportant within the secluded world of the residential aged care facility, and they described conflicts regarding how they wanted things to be done. For instance, staff made decisions on behalf of the older persons, without asking them first, which was experienced as humiliating by the older persons. This is visualised by the quotation below.

*“Julia: You don’t get the feeling that anyone cares here. No. It’s a ‘let-it-go’ mentality, like it’s not my job.*

*Interviewer: No, okay, mmm, I understand.*

*Julia: And that, they think this is a home, it’s our home, and it should be pleasant when people come in here.”*

Julia, 94 years

In addition, some of the staff members were experienced to have a let-go-mentality, which led to feelings of both insecurity and inferiority among the older persons. For instance, the older persons were unsure whether staff would respond on alarms, as shown by this quotation:

“*Interviewer: Do they not come when you press the alarm?*

*Julia: No, no, no, no, I can, one calls and calls and calls, (they) do not come.*”

Julia, 94 years

***Infringement of integrity:*** Integrity was described in terms of having power over one’s own personal and undisturbed place and having control over it. This was a foundation for the feeling of home, but the older persons’ power was infringed by staff and neighbours who, at times, entering the apartment without knocking first or staff moving the older persons’ belongings without asking. Our interpretation was that this infringement of integrity was a transgression on the person’s feeling of power over their influence of their everyday life and home, where one should be at most ease and be able to gather strength. The following quotation is an example of the infringement of integrity:

“*Thomas: we had one (person) who lived here before.*

*Interviewer: Yes*

*Thomas: And I came in, and he stood there poking about in my papers.*

*Interviewer: Mmm, yes*

*Thomas: and then he did not want to go out, so then he wanted to start a fight.*”

Thomas, 79 years

The older persons were also advised by staff not to keep valuables (both economically and emotionally valuable) in their apartments due to the risk of them being stolen. This meant that the older persons’ feeling of safety and their perception of themselves as inviolable persons was disturbed. The restrictions imposed by the COVID-19 pandemic further challenged the older persons’ integrity as they were experienced as unclearly communicated and difficult to understand. Decisions on how to minimise the spread of COVID-19 were being made about the older persons instead of together with them. For instance, the restrictions meant that visits from family and friends, and activities and services such as bingo and pedicure care, ceased without explanation, which affected the older persons’ opportunities to influence their everyday life in a negative way.

#### The importance of dialogue.

This core theme describes the importance of having the opportunity to hold meaningful dialogues with staff and neighbours as part of everyday life. Recurring and systematic dialogues were described as necessary for the older persons to get their needs met and feeling that they were important for staff (as visualised by quotation below). Yet, the older persons experienced that staff talked to them primarily as a function to be able to carry out their work, and the institutional cultures were experienced to reduce the older persons’ opportunities to make their voices heard regarding what should be done, how, and when. They did not always feel sufficiently informed about what would happen, and they experienced difficulties with getting assistance and support when needed. Some staff members were very responsive, whereas others were not experienced as attentive to the older persons’ stories, meaning that the older persons experienced both disharmony and harmony in dialogues between themselves and the staff. In relation to neighbours, the older persons expressed a desire for meaningful dialogues with peers, but this could be difficult due to their varying degrees of cognitive ability and insufficient arenas for social interaction in the facilities. As a result, the older persons isolated themselves in their apartments to a greater extent than desired. The importance of dialogue is further described in the subthemes: *The surrounding as a catalyst for the person’s life-story* and *Becoming part of a community.*

*“He, uh, we have certain tasks he (staff member) would do, maybe make the bed, and in the meantime, I usually shower. I shower by myself, but I like to have someone in the room, and then he takes the opportunity to clean and change the towels and the bed, and so on. And if he’s away, which he often is because of small children, he takes care of them when they’re sick. And then there’s no one else to take over.”*

Julia, 94 years

***The surrounding as a catalyst for the person’s life-story*:** The importance of being surrounded by flowers, nature, and a garden was expressed as having something beautiful to look at, but also as a means to influence everyday life by being a catalyst for the older persons’ memory of their lives before they moved to the facility, i.e., their life story. Music was also something that sparked the older persons’ memories. These catalysts were experienced as very important as they reminded them of times passed and provided both consolation and a basis for dialogues with staff. The older persons also kept artefacts such as paintings, post cards and other objects in their apartments to remind themselves and others about their personality and life story (visualised by the quotation below). Social networks outside the facility, such as relatives and friends, was another way of sparking the older persons’ life story. Concerns with COVID-restrictions increased the older persons’ issues with technology to keep contact with the outside world, or not knowing who to turn to for technological support. This was experienced as barriers for maintaining the never-ending creation of the life story. Some of the older persons therefore communicated with relatives and friends through post and telephone, as a complement to modern information technology such as tablets and computers.

*“John: There are simply many memories associated with these stones. Yes, it’s memories and camaraderie. The camaraderie is perhaps even more important.”*

John, 83 years

***Becoming part of a community*:** This subtheme concerns opportunities to influence everyday life through becoming part of a community at the facility, as well as maintaining a connection to the society. Organised joint activities at the facility were experienced as one way of becoming a part of a community, provided that the activities matched the older persons’ interests and facilitated dialogues with neighbours and/or staff. The older persons stressed the importance of dialogues with them in the planning of activities, to make sure that they could influence the selection of activities to choose from. They also described a wish that the cancellation of activities due to COVID-19 restrictions would have been discussed with them before being implemented. One activity that was expressed as especially meaningful to engage in, and that was not affected by the restrictions, was meals, as it meant that the older persons could find their place within the facility’s community through dialogues with other persons around the table. They also expressed a wish to be involved in dialogues regarding the meals to influence everyday life, for instance by choosing types of food as well as how and when it was served. The existing food council in one of the facilities was, however, experienced as tokenistic, with no real influence over the selection of meals, as described by one of the involved persons:

*“Interviewer: Do you get any response for these complaints on the food?*

*Lisa: No, now there are food, two (persons) are food councils here. And we have tried to say to them that it, it does not seem to work. And also, she who handles the food, has had a meeting with a few weeks, or months in between. And I have only been there once, then during spring before Corona came, but my friend has been there three or four times. And nothing ever happens.*

*Interviewer: No*

*Lisa: No. There is no change.”*

Lisa, 89 years

In addition, becoming a part of a community involved maintained opportunities to feel connected to the larger community, outside the facility, for instance through newspapers and magazines. However, issues with visual impairments meant that some of the older persons needed someone to read out loud for them.

## Discussion

The aim with this study was to identify and elucidate frail older persons’ opportunities to influence their everyday life in residential aged care facilities. A major finding was the identification of the opposite, the older persons felt inferior to the staff and the institutional cultures limited their opportunities to influence their everyday life. The dynamic exchange between a game of power and the importance of dialogue with staff is supported by earlier research [47], stating that the older persons’ voices are heard if they do not interfere with the operation of the facility [47]. We define culture as a set of norms and practices that are shared [48], and norms are understood through Goffman [49], as unexpressed rules that heavily influence how people act, think, and self-adjust. In this study, norms and practices were shared among persons working in the facility, but not with the persons living in the facility. In relation to this, institutional cultures are regarded as a widened “local routine culture” [50, p.36] that has a major impact on the older persons’ opportunities to make choices in leisure and social activities [23]. Complemented by our findings, this suggests that improved opportunities to engage in different types of activities and having the possibility to choose these activities, could make life in residential aged care facilities more fulfilling than they currently are.

Both the opportunities of making activity choices and how culture influences the everyday life of frail older persons have bearing on PCC. In the present study, the institutional cultures of the residential aged care facilities seemed to be influenced by a more or less pronounced PCC as defined by Ekman et al. [51]. This meant that the opportunities for staff to listen to and acknowledge (through for example documentation) the older persons’ narratives seemed to vary both within and between the facilities, affecting the building of partnerships between older persons and staff. This is reflected in the visualisation of how the frail older persons described their opportunities to influence their everyday lives in relation to which persons who were at work, when and how they worked, how the organisation managed resources and who lived in the facility. This is also described in Keane et al. [23] where the cultural environment had a substantial effect on the opportunities for persons living in residential aged care facilities to choose which activities to engage in [23].

The influence of institutional cultures on both persons living and working in residential aged care facilities has been described in previous literature [52,53]. For example, Lood et al. [52] and French [53] visualise how institutional cultures that emphasise operational procedures can constrain and alienate staff from fulfilling various aspects of their caregiving roles. French [53] highlights how such cultures can result in a loss of skills and competence, reflecting the impact of limited opportunities for older persons to engage in meaningful activities on staff. Lood et al. [52], further illustrate the importance of a person-centred culture for persons working in residential aged care facilities to feel that they can become part of an upward spiral of performing person-centred actions and being person-centred as a team together with the older persons [52]. Previous research [54] also suggests that PCC needs to be both practically and organisationally created in dialogue with persons in need of care and support and persons who provide care [54]. This study visualises examples of how such dialogues may be deficient or even lacking in residential aged care facilities, expressed through the experience of a game of power and difficulties with dialogue between the older persons and the persons working in the facility. It is therefore important to address factors, such as social power relations, that may hinder person-centred doing and being among persons working in residential aged care facilities [52].

Person-centred doing means adopting a person-centred approach (person-centred being) [54] both towards the person who is receiving care but also towards colleagues at the workplace, to create a person-centred culture. A concept that is close to the notion of person-centred culture is person-centred climate. It can be characterised by the feeling of everydayness, hospitality, and safety, where the residential aged care facility offers a physical environment of beautiful views, flowers, and the opportunity to socialise among neighbours in a relaxed home-like environment [55]. In relation to the findings of this study, a person-centred climate could be understood as an environment where the older persons both feel at home and seen by other persons working and living in the facility. It could also be understood as a culture that provides opportunities for social activities and to feel safe because persons working in the facility are available for dialogues on everyday matters. This means that persons working in residential aged care facilities should be able to take the time to listen to each person and, in dialogue, create something that works for both the older person and them as staff members. This should then be documented as a natural part of the everyday work as a way of taking the older persons seriously and to attribute them as capable of making choices.

In the context of person-centred doing and being, taking frail older persons seriously and regard them as capable to be involved in everyday life can be seen as a care approach grounded on Ricoeur’s [56] little ethics. That is, “…aiming at the “good life” with and for others, in just institutions” [57, p.172]. Aiming at the good life, or the flourishing life, refers to what is considered as good according to an Aristotelean and Kantian ethical view. With and for others, requires a reversibility regarding those involved in terms of roles but also reciprocity to understand what is considered a good life and for whom [56]. We interpret this as a need for persons working in residential aged care facilities to strive for an approach that allows them to see themselves in the older persons, to be able to take them seriously and attribute responsibility to them, in the same way that they, as staff members, disclaim responsibility. Just institutions refer to institutions where each person’s concerns and life story are considered, making the person capable within the institution [56]. Applying our findings, and Ricoeur’s [56] little ethics to practice, increasing frail older persons’ feelings of being capable in residential aged care facilities could be exercised through the implementation of PCC [58] and a person-centred climate [55], to increase the older persons’ opportunities to influence their everyday life and to make choices more equitable [59].

Being able to influence choices in terms of what one does, when, how and where they are done could be seen as a way of existing, or taking a place in the world, as well as being able to live a good life, with and for others. Our interpretation of the older persons’ narrations was that talking to the older persons beyond physical needs was not experienced as being part of the working routines at the facilities. Possibly because the persons working there did not have the structural and organisational conditions to work together with the persons living there, i.e., the culture was not person-centred enough. This study contributes with knowledge on activity constraints in residential aged care facilities, and addresses some of the mechanisms behind these constraints. For instance, our findings confirm previous research [60] that has shown that councils and groups, which were supposed to increase involvement and meaningful dialogue with the older persons, had no actual influence, but were rather seen as a kind of window dressing or tokenism [61]. As visualised by Harnett [50], few previous studies have explored such negative influence of power in residential aged care facilities [50]. Power is maintained in the dynamic interaction between people [62], and in our study, the older persons’ feelings of inferiority can be interpreted as an expression of normalising power [63], where the older persons submit to the order of power when it has become a norm. It is possible that counterpower arises in such situations, which in this study manifested itself as the older persons sometimes withholding information from staff. Withholding information was a way of reducing the feeling of powerlessness, i.e., a kind of counterpower [62] that gave the older persons a sense of control over a certain situation.

### Methodological limitations

As persons in most countries live longer and populations get older, there is a need for research involving older persons living with frailty and/or neurocognitive disorders in research on ageing and health [64]. Arguably, research with these groups of people can be considered unethical, but it could be equally considered unethical not listening to them and making use of their knowledge that could benefit frail older people as a group. Hammell [65] has highlighted the importance of adopting a capability approach to research to put focus on the genuine opportunities each person has to leverage their abilities and lead the lives they have a reason to value [59]. Engaging in research-related activities, such as the photographic sessions and interviews in this study, have been described as meaningful for frail older persons [6,66]. However, power dynamics between persons who are researchers and frail older persons need to be reflected on before, during, and after a study [66]. As described in our previous study [6], the photo-elicitation interviews meant that the older persons had the opportunity to challenge their ageing bodies and institutional cultures to exercise power over the data generation [6]. This challenges ageist assumptions frequently hidden in society [67], and the photo-elicitation interview method was chosen to provide opportunities for older persons with frailty and/or neurocognitive disorders to leverage their abilities to make their voices heard, beyond merely using words. Methodological aspects are presented in another article [6], concluding that the method reduced institutional cultures’ influence on the older persons’ participation in research. Although the method has limitations, such as need for help taking photos, the method could be considered as a means to involve persons living in residential aged care facilities in research.

The COVID-19 pandemic induced some limitations, with restricted opportunities for the researchers to be part of the involvement of older persons in the study. As such, the gatekeeper role of the residential aged care staff may have caused an indirect exclusion of older persons who they did not assess as capable or eligible to be involved in the study. Elaborated on further in another publication [6], we want to highlight the impact staff as gatekeepers may have in the involvement of persons living in residential aged care facilities, while also often being the only way of contacting this group of people. Moreover, it was not possible to control when the interviews were conducted, which meant that some of them were conducted several weeks after the data collection. Our previous article [6] indicates that the period between photos being taken and the interviews did not have a major influence on the older persons’ narrations since their cognitive impairments influenced their memory regardless of time between the photo session and the interview.

### Implications

This study offers valuable knowledge for levelling frail older persons’ opportunities to influence everyday life and choose which activities to engage in, providing a better foundation for how to support a rich and engaged life in residential aged care facilities. Based on our findings, we recommend that older persons living in residential aged care facilities are acknowledged ass having the capabilities needed to contribute to co-creation of new knowledge if they are given the opportunity [68]. Power needs to be balanced between older persons and staff to support PCC within institutional cultures to give justice to frail older persons as capable of contributing with valuable knowledge, even if physical and cognitive impairments are present. On a more methodological note, we agree with Hammell [65] that future research should adapt a capability approach to research to identify what is needed to support older persons’ participation and involvement in research on ageing and health.

## Conclusion

This article confirms previous findings about everyday activities [20,46] and contributes with the illustration of experiences from persons living in residential aged care facilities with neurocognitive disorders who have often been unrightfully excluded from research. Our main conclusion is that the frail older persons in this study experienced that their opportunities to influence their everyday life was limited by institutional cultures, and that the cultures were permeated by the scarcity of meaningful dialogue and a game of power. There were few clear examples of person-centred doing and being, meaning that the older persons’ abilities and needs were not always acknowledged by staff. They did not always feel as equal partners in the care or in their activities choices as part of everyday life, uncovering that a person-centred climate is not always achieved. This is interesting, especially since Swedish legislation [3–5] obliges residential aged care staff to work in a person-centred way. Our study also indicates power, dialogues, and institutional cultures as important areas of focus when developing PCC in residential aged care facilities, to go from an institutionalised culture to a person-centred culture. The findings could be used to spark an incentive to increase awareness of, and/or competence in, the importance of power and dialogue for the implementation of a PCC and person-centred climate in residential aged care facilities. We encourage future researchers to further explore the opportunities for persons living in residential aged care facilities, with or without neurocognitive disorders, to participate in studies elucidating their wishes and needs. Further studies are also needed with people working in these facilities exploring their opportunities to work in a person-centred way.

## Abbreviations

WHO: World Health Organization
SDG: Sustainable Development Goals
PCC: Person-centred care
GPCC: Gothenburg university centre for Person-Centred Care
DSM5: Diagnostic and Statistical Manual of mental Disorders 5
RTA: Reflexive Thematic Analysis.
